# Placebo response in treatment resistant depression: a systematic review and meta-analysis of multiple treatment modalities

**DOI:** 10.1192/bjo.2021.697

**Published:** 2021-06-18

**Authors:** Brett D M Jones, Cory R. Weissman, Jewel Karbi, Tya Vine, Louise S. Mulsant, Benoit Mulsant, Andre Brunoni, M. Ishrat Husain, Lais B. Razza, Daniel Blumberger, Zafiris J Daskalakis

**Affiliations:** 1 University of Toronto, Department of Psychiatry; 2 McMaster University; 3 Nutrition and Food, Nova Scotia Health Authority; 4 University of Toronto, Department of Psychiatry, Centre for Addiction and Mental Health; 5 Department of Internal Medicine, Faculty of Medicine, University of Sao Paulo, Laboratory of Neurosciences (LIM-27), Instituto Nacional de Biomarcadores em Neuropsiquiatria (INBioN), Department and Institute of Psychiatry, Faculdade de Medicina, Universit; 6 Centre for Addiction and Mental Health, University of Toronto, Department of Psychiatry; 7 Department of Psychiatry, University of California San Diego

## Abstract

**Aims:**

The placebo response in depression clinical trials is a major contributing factor for failure to establish the efficacy of novel and repurposed treatments. However, it is not clear as to what the placebo response in treatment-resistant depression (TRD) patients is or whether it differs across treatment modalities. Our objective was to conduct a systematic review and meta-analysis of the magnitude of the placebo response in TRD patients across different treatment modalities and its possible moderators.

**Method:**

Searches were conducted on MEDLINE and PsychInfo from inception to January 24, 2020. Only studies that recruited TRD patients and randomization to a placebo (or sham) arm in a pharmacotherapy, brain stimulation, or psychotherapy study were included (PROSPERO 2020 CRD42020190465). The primary outcome was the Hedges’ g for the reported depression scale using a random-effects model. Secondary outcomes included moderators assessed via meta-regression and response and remission rate. Heterogeneity was evaluated using the Egger's Test and a funnel plot. Cochrane Risk of Bias Tool was used to estimate risks.

**Result:**

46 studies met our inclusion criteria involving a total of 3083 participants (mean (SD) age: 45.7 (6.2); female: 52.4%). The pooled placebo effect for all modalities was large (N = 3083, g = 1.08 ,95% CI [0.95-1.20)I ^2^ = 0.1). The placebo effect in studies of specific treatment modalities did not significantly differ: oral medications g = 1.14 (95%CI:0.99-1.29); parenteral medications g = 1.32 (95%CI:0.59-2.04); ayahuasca g = 0.47 (95%CI:-0.28-1.17); rTMS g = 0.93 (95%CI:0.63-1.23); tDCS g = 1.32 (95%CI:0.52-2.11); invasive brain stimulation g = 1.06 (95%CI:0.64-1.47). There were no psychotherapy trials that met our eligibility criteria. Similarly, response and remission rates were comparable across modalities. Heterogeneity was large. Two variables predicted a lager placebo effect: open-label prospective design (B:0.32, 95%CI: 0.05-0.58; p:0.02) and sponsoring by a pharmaceutical or medical device company (B:0.39, 95%CI:0.13-0.65, p:0.004)). No risk of publication bias was found.

**Conclusion:**

The overall placebo effect in TRD studies was large (g = 1.08) and did not differ among treatment modalities. A better understanding of the placebo response in TRD will require: standardizing the definition of TRD, head-to-head comparisons of treatment modalities, an assessment of patient expectations and experiences, and standardized reporting of outcomes.

